# The corticospinal tract in multiple sclerosis: correlation between cortical excitability and magnetic resonance imaging measures

**DOI:** 10.1007/s00702-024-02849-0

**Published:** 2024-10-17

**Authors:** Paul Kauv, Moussa A. Chalah, Alain Créange, Jean-Pascal Lefaucheur, Jérôme Hodel, Samar S. Ayache

**Affiliations:** 1 Service de Neuroradiologie, Hôpital Henri-Mondor, Assistance Publique- Hôpitaux de Paris (AP-HP), 51 avenue du Maréchal de Lattre de Tassigny, Créteil Cedex, 94010 France; 2 EA 4391, Excitabilité Nerveuse et Thérapeutique, Université Paris-Est-Créteil, Créteil, France; 3 Department of Neurology, Gilbert and Rose-Marie Chagoury School of Medicine, Lebanese American University, Byblos, Lebanon; 4 Institut de la Colonne Vertébrale et des NeuroSciences (ICVNS), Centre Médico-Chirurgical Bizet, Paris, France; 5 Institut de Neuromodulation, Pôle Hospitalo-Universitaire Psychiatrie Paris 15, GHU Paris Psychiatrie et Neurosciences, Hôpital Sainte-Anne, Paris, France; 6 Service de Neurologie, Hôpital Henri-Mondor, Assistance Publique- Hôpitaux de Paris (AP-HP), Créteil, France; 7 Department of Clinical Neurophysiology, DMU FIxIT, Henri Mondor University Hospital, Assistance Publique- Hôpitaux de Paris (AP-HP), Créteil, France; 8 Department of Radiology, Groupe Hospitalier Paris Saint-Joseph, Paris, France; 9 Centre d’Imagerie Médicale Léonard de Vinci, Paris, France

**Keywords:** Cortical excitability, Transcranial magnetic stimulation, Corticospinal tract, Diffusion tensor imaging, Double inversion recovery, Phase sensitive inversion recovery

## Abstract

Multiple sclerosis (MS) is a central nervous system disease involving gray and white matters. Transcranial magnetic stimulation (TMS) and magnetic resonance imaging (MRI) could help identify potential markers of disease evolution, disability, and treatment response. This work evaluates the relationship between intracortical inhibition and facilitation, motor cortex lesions, and corticospinal tract (CST) integrity. Consecutive adult patients with progressive MS were included. Sociodemographic and clinical data were collected. MRI was acquired to assess primary motor cortex lesions (double inversion and phase-sensitive inversion recovery) and CST integrity (diffusion tensor imaging). TMS outcomes were obtained: motor evoked potentials (MEP) latency, resting motor threshold, short-interval intracortical facilitation (ICF) and inhibition. Correlation analysis was performed. Twenty-five patients completed the study (13 females, age: 55.60 ± 11.49 years, Expanded Disability Status Score: 6.00 ± 1.25). Inverse correlations were found between ICF mean and each of CST radial diffusivity (RD) (ρ =-0.56; *p* < 0.01), CST apparent diffusion coefficient (ADC) (ρ=-0.44; *p* = 0.03), and disease duration (ρ=-0.46; *p* = 0.02). MEP latencies were directly correlated with disability scores (ρ = 0.55; *p* < 0.01). High ADC/RD and low ICF have been previously reported in patients with MS. While the former could reflect structural damage of the CST, the latter could hint towards an aberrant synaptic transmission as well as a depletion of facilitatory compensatory mechanisms that helps overcoming functional decline. The findings suggest concomitant structural and functional abnormalities at later disease stages that would be accompanied with a heightened disability. The results should be interpreted with caution mainly because of the small sample size that precludes further comparisons (e.g., treated vs. untreated patients, primary vs. secondary progressive MS). The role of these outcomes as potential MS biomarkers merit to be further explored.

## Introduction

Multiple sclerosis (MS) is a multifocal disease of the central nervous system that involves white matter as well as grey matter (Geurts & Barkhof, 2008). Progressive MS types are associated with important clinical disability despite the moderate lesion burden reported using conventional magnetic resonance imaging (MRI) (Miller and Leary 2007). This suggests other mechanisms at the basis of disability (e.g., cortical lesions, alteration of corticospinal tract (CST) function and integrity) (Rovaris et al. 2000; Kutzelnigg et al. 2005; Geurts & Barkhof, 2008; Roosendaal et al. 2009).

Transcranial magnetic stimulation (TMS) is a non-invasive neurophysiological technique that allows the exploration of cortico-nuclear and cortico-spinal connections using motor-evoked potentials (MEP) (Rossini et al. 2015). TMS has been applied in the context of MS aiming to identify potential biomarkers of the underlying disease processes, disease evolution, or clinical response to treatments (Chalah et al. 2020, 2021). For instance, using single-pulse paradigms, abnormalities were previously described in patients with MS (PwMS) compared to healthy subjects, including prolonged central motor conduction time (Cowan et al. 1984; Mills and Murray 1985; Ravnborg et al. 1991) and increased resting motor threshold (rMT) (Zipser et al. 2018). Prolonged latencies were also reported and appear to be directly correlated with the expanded disability status scale scores (EDSS) (Schmierer et al., 2002; Kale et al. 2009). In addition, prolonged latencies and low MEP amplitudes, along with higher central motor conduction time and low cortical excitability (i.e., rMT), were previously correlated with demyelination and axonal loss in PwMS (Stampanoni Bassi et al. 2020). Also, corticomuscular latency seems to worsen over a two-year follow-up in PwMS suffering from progressive types, with a neurophysiological deterioration observed at one year before the appearance of clinical aggravation (Hardmeier et al. 2020). Moreover, TMS measures obtained using paired-pulse paradigms could help assess cortical excitability. Here, intracortical inhibition and facilitation measures were found to be associated with disability (Vucic et al. 2012; Mori et al. 2013; Simpson and Macdonell 2015; Nantes et al. 2016a; Neva et al. 2016), relapses (Caramia et al. 2004), inflammation (Rossi et al. 2015), disease progression (Ayache et al. 2015), or response to treatments (Mainero et al. 2004; Créange et al. 2013; Ayache et al. 2014; Landi et al. 2015; Russo et al. 2015) (for review see Stampanoni Bassi et al. 2020).

Besides TMS, magnetic resonance imaging (MRI) constitutes another way to explore primary motor cortex lesions and CST abnormalities. 3D double inversion recovery (DIR) which suppresses both cerebrospinal fluid and white matter signals, and phase-sensitive inversion recovery (PSIR) which improves resolution and contrast of white and grey interface, are the most sensitive and specific sequences to detect motor cortical lesions in PwMS (Geurts et al. 2005; Wattjes et al. 2007; Simon et al. 2010; Sethi et al. 2012, 2013; Vural et al. 2013; Favaretto et al. 2015). Diffusion tensor imaging (DTI) with tractography is another MRI sequence that allows identifying white matter fiber trajectory (e.g., CST) and providing information about integrity along these tracts with DTI-derived measures). In this context, DTI has been applied in PwMS showing a decreased fractional anisotropy compared to healthy controls (Wilson et al. 2003; Lin et al. 2007; Gorgoraptis et al. 2010; Pawlitzki et al. 2017). The latter finding was correlated with primary motor cortex thinning and high expanded disability status scale (EDSS) scores (Bergsland et al. 2015; Tovar-Moll et al. 2015).

Until now, very few studies have combined TMS and MRI data and suggested the pertinence of this approach in providing complementary information and gaining insight into the relationship between neurophysiological and neuroanatomical outcomes. Such a strategy can assess the function and microstructure respectively. Therefore, the main objective of this work was to evaluate the relationship between intracortical inhibition and facilitation measures, the number of lesions within the primary motor cortex detected by DIR/PSIR, and DTI-based measures of CST. In addition, the current study aimed to assess the relationship between these outcomes, and sociodemographic and clinical data.

## Methods

### Study participants

Patients were consecutively recruited from the neurology department at Henri Mondor Hospital, Creteil, France. Inclusion criteria were (i) age from 18 to 75 years, (ii) having a confirmed diagnosis of MS according to McDonald criteria (Thompson et al. 2018), and (iii) suffering from a primary progressive (PPMS) or a secondary progressive form (SPMS) of the disease (Lublin et al. 2014), (iv) being free from relapse and (iv) having a stable pharmacological treatment over the last three months. Exclusion criteria included (i) a history of seizures, (ii) the presence of any ferromagnetic implant, or (iii) the absence of MEP. Patients had MRI and TMS within the same month. Ethical committee approval was obtained, and patients gave their written informed consent prior to participation.

### Sociodemographic and clinical data

A detailed medical history and data from neurological examination were collected and the EDSS scores were calculated (Kurtzke 1983). Particular attention was paid to any new or deterioration of neurological deficit. Age, sex, disease duration, and type, and treatments were collected.

### Neurophysiological data

The method has been previously described (Chalah et al. 2018, 2019, 2020). Briefly, TMS was performed using Magstim 200 (Magstim Co., Carmarthenshire, UK). A circular coil was used to deliver the stimulations (90 mm coil type P/N 9784- 00; Magstim Co., Whitland, Carmarthenshire, UK). The coil was positioned over the vertex with its handle oriented posteriorly. A cap was used to mark the coil position to ensure it was stable throughout the session. MEPs were recorded at rest over the first dorsal interosseous (FDI) hand muscle of the most affected hand or hemibody using self-adhesive surface electrodes positioned in a belly-tendon montage (Ref. 86 9013S0242, Natus-Dantec, Skovlunde, Denmark). TMS was applied over the corresponding contralateral primary motor cortex (Ayache et al. 2014, 2015). Different coil sides (A or B) were used for a predominant stimulation of the motor cortex side (left or right, respectively). Phasis II machine (EsaOte, Florence, Italy) was used for recording the electromyographic (EMG) signals which were bandwidth filtered (20 Hz − 2 kHz) and amplified (50–500 µV/division). Auditory feedback of the EMG signal was used to ensure muscle relaxation.

First, the rMT was determined using a step width of 1% of the maximal stimulator output. It corresponds to the stimulation intensity capable of inducing MEP of more than peak-to-peak amplitude 50 µv in at least 5 out of 10 trials (Rossini et al. 1994, 2015; Rothwell et al. 1999). Second, short-interval intracortical inhibition (SICI) and facilitation (ICF) were studied by adopting a double-pulse paradigm using two monophasic Magstim 200 stimulators connected via a Bistim module. The “test” (conditioning) and “conditional” (suprathreshold) stimulations had respective intensities of 80% and 120% of the rMT, and the adopted interstimulus intervals (ISI) were 2 and 4 ms for SICI, and 10, 12 or 15 ms for ICF (Kujirai et al. 1993). Minimal latencies were calculated from eight trials of unpaired control test stimuli recorded at 120% of the rMT.

Afterward, for each ISI, four trials of paired stimuli were recorded and averaged. Calculating the percentages of SICI and ICF relied on the MEP Peak-to-peak amplitudes obtained following paired stimuli to those of control MEPs. Results were expressed as the amount of inhibition (SICI = 100% – paired/control MEP%) and facilitation (ICF = paired/control MEP% – 100%). Mean and maximal percentages of SICI and ICF were considered (ICImean, SICImax, FICmean, FICmax) (Lefaucheur 2012, 2016).

### MRI procedure

MR examinations were performed at 3T (Skyra, Siemens, Erlangen, Germany) equipped with a 64-channel head coil. The imaging protocol included: a DIR (TR/TE/TI/TI2:7500/317/3000/400ms, matrix:192 × 174, voxel size:0.7 × 0.7 × 0.8 mm, number of slices:192, time 7 min), PSIR (TR/TE/TI: 2740/9.9/400ms, matrix:384 × 269, voxel size:0.6 × 0.6 × 3 mm, number of slices:34, time 4:30 min) and a 12-directions DTI sequence at a b value of 800 s/mm² (TE/TR:91/6800ms, matrix:122 × 124, voxel size:2 × 2 × 2.5 mm, number of slices:50, time 4:30 min).

### Image analysis

Cortical lesions of the primary motor cortex from the contralateral side of TMS measurements side were identified and numbered on DIR and PSIR images according to previous recommendations (Sethi et al. 2012, 2013; Geurts et al. 2011). Special attention was also paid to precentral knob lesions which were also separately identified and numbered (Yousry et al. 1997) (typical shape of the hand motor area).

DTI data were reconstructed using the Diffusion Toolkit to create 3D images of the WM fiber tracts in the TrackVis program (www.trackvis.org) (Wedeen et al. 2008), generating fractional anisotropy (FA), apparent diffusion coefficient (ADC), and the three eigenvalues of the diffusion tensor maps. A track angle threshold of 30° was used. Tractography of CST from the contralateral side regarding TMS measurement side was performed by manually drawing region of interest (ROI) on each subject FA color axial map of the posterior internal capsule region and CST tracts were confirmed visually for anatomic accuracy. Six measurements were obtained using the TrackVis software package for each CST tract: volume, mean FA, mean ADC, axial diffusivity (AD) and radial diffusivity (RD). AD corresponded to the first eigenvalue (i.e., largest eigenvalue) while RD was defined as the average of the second and third eigenvalues of the diffusion tensor. MRI rater (P.K.) was blinded to clinical and neurophysiological data.

### Statistical analysis

Non-parametric tests were applied since not all data passed the normality test using the Kolmogorov-Smirnov method. Spearman rank correlations (ρ) were calculated between TMS data (SICI_mean_, SICI_max,_ FIC_mean_, FIC_max_, RMT, and MEP latencies) and MRI data (number of cortical lesions of primary motor cortex and precentral knob; FA, ADC, AD, RD, volume of CST) using Prism (GraphPad). Correlations were computed according to TMS measurement side (e.g., TMS recordings from the right FDI muscle were correlated to MRI cortical lesions and CST from the left side). We also assessed the correlation between TMS and MRI measures with sociodemographic and clinical data (age, EDSS and disease duration). Mean ± standard deviation (SD) of all data was presented. *P* < 0.05 was considered as significant.

## Results

### Sociodemographic and clinical data

Twenty-nine consecutive patients were assessed for eligibility. Four patients were not included for various reasons: did not accept to participate (*n* = 2), recent stroke (*n* = 1), and recent diagnosis of cancer (*n* = 1). 25 patients were enrolled and completed the study. There were 12 males and 13 females, with a mean age of 55.60 ± 11.49 years, a mean disease duration of 14.20 ± 5.99 years, and a mean EDSS score of 6.00 ± 1.25. Thirteen patients had PPMS and twelve had SPMS. Their treatments were as follows: Dimethyl fumarate (*n* = 5), interferon-β (*n* = 2), steroids pulse therapy (*n* = 2), teriflunomide (*n* = 2), rituximab (*n* = 1), methotrexate (*n* = 1), fingolimod (*n* = 1). Eleven were not receiving MS medications at the time of the study. Demographic data are summarized in Table 1.

**Table 1:** Clinical and sociodemographic data of patients with multiple sclerosis

Variables	Data
Male/Female (number)	12/13
Age (years)	55.60 ± 11.49
Disease duration (years)	14.20 ± 5.99
EDSS (score)	6.00 ± 1.25
Immunomodulatory drugs	Dimethyl fumarate (*n* = 5), interferon-β (*n* = 2), steroids pulse therapy (*n* = 2), teriflunomide (*n* = 2), rituximab (*n* = 1), methotrexate (*n* = 1), fingolimod (*n* = 1), none (*n* = 11)

Values are expressed in mean ± SD, number, or score. EDSS: expanded disability status scale

### Neurophysiological and neuroimaging data

**Fig. 1:**
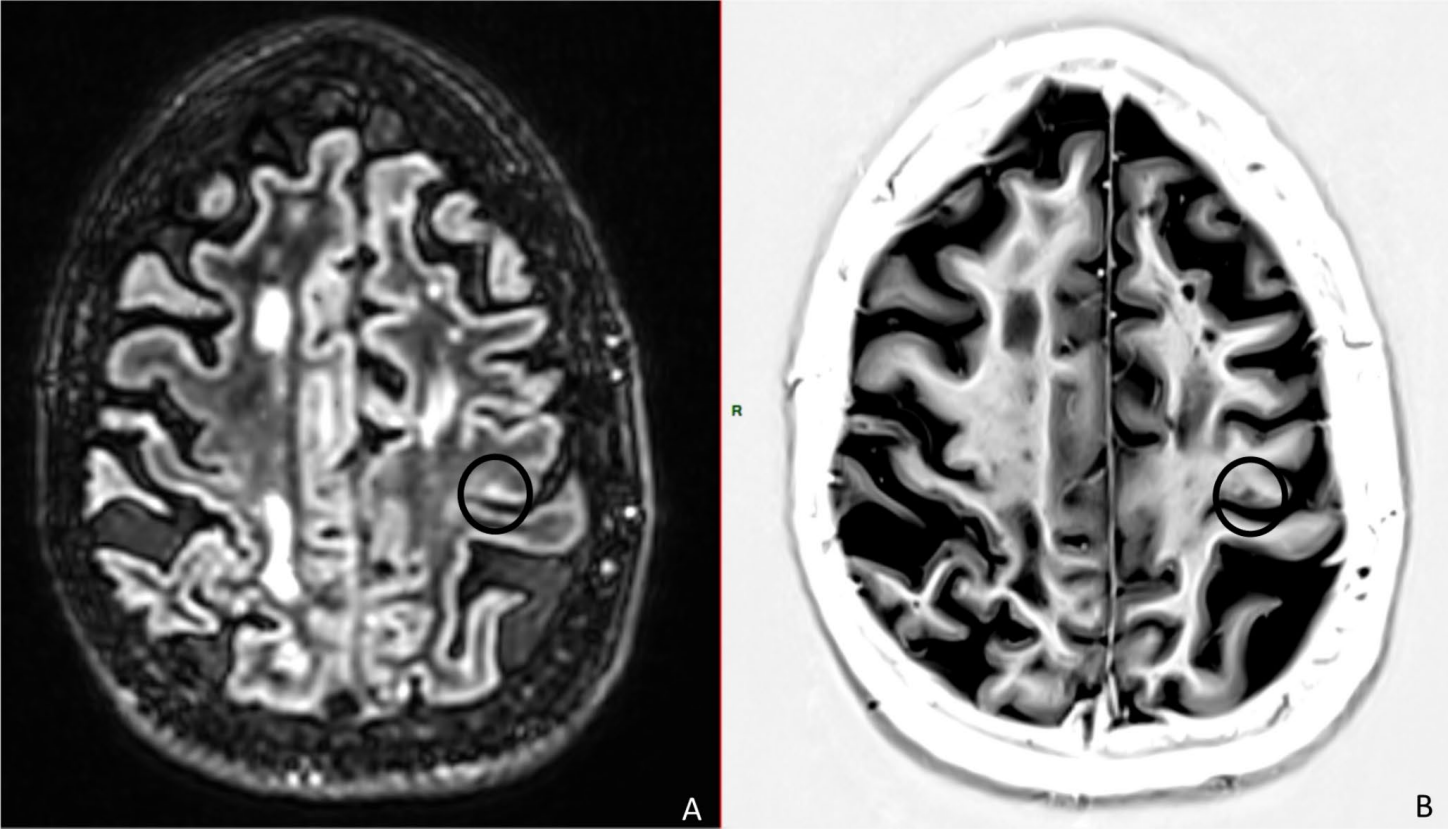
Example of neuroimaging obtained in a 67-year-old patient with primary progressive multiple sclerosis showing one cortical lesion in knob (circle) hyperintense on 3D double inversion recovery (DIR) image (**A**) and hypointense in phase-sensitive inversion recovery (PSIR) image (**B**)

For the 25 patients, the total number of motor cortical lesions was 11 (lesions present in 5 patients), and the number of precentral knob lesions was 4 (lesions present in 4 patients) (example in Fig. 1).

**Fig. 2:**
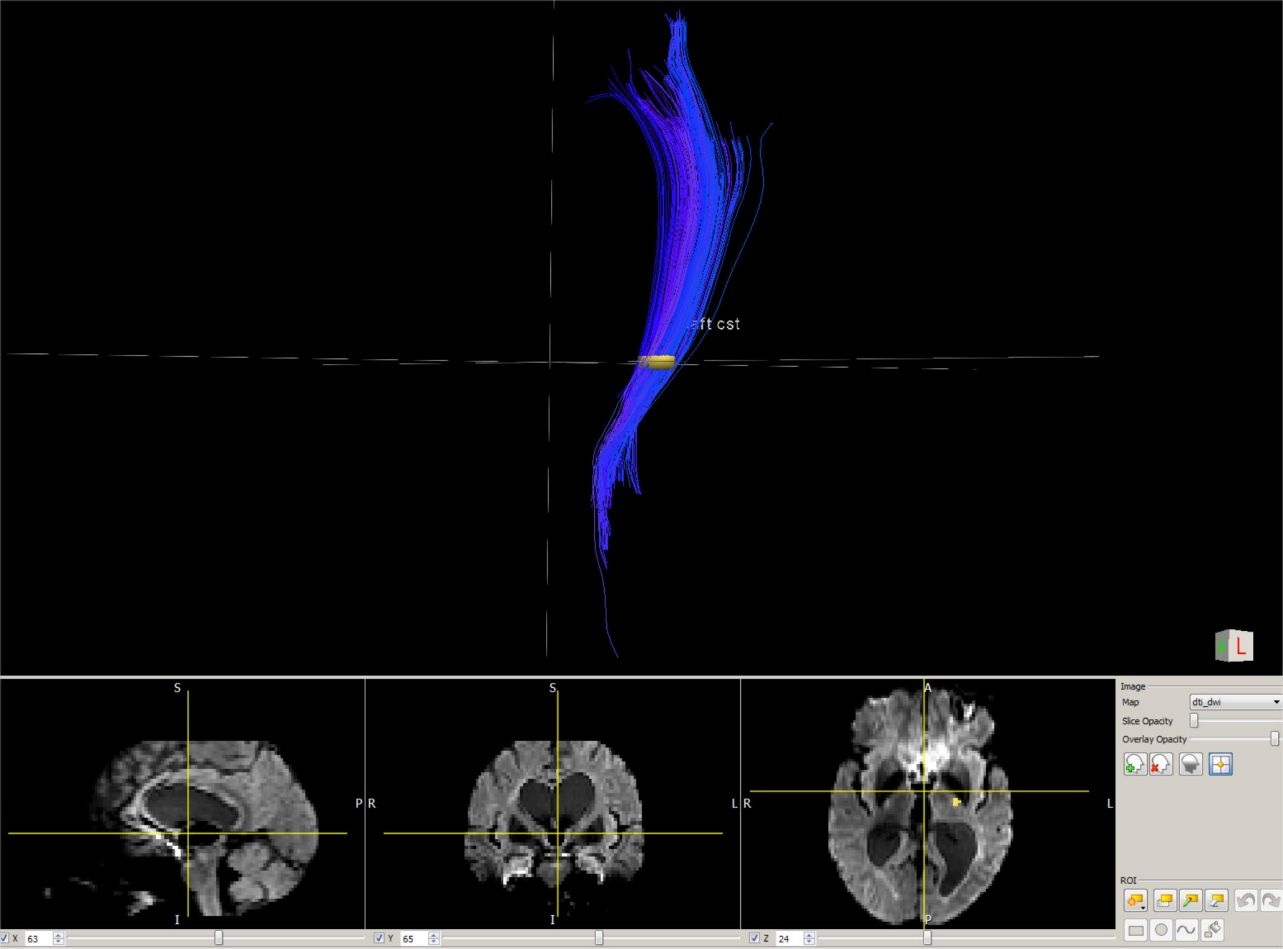
Example of magnetic resonance imaging tractography of left corticospinal tract (CST) (blue, upper image) performed from posterior internal capsule ROI (yellow cross, the lower images) in a 43-year-old patient with secondary progressive multiple sclerosis. The measures were as follows: fractional anisotropy (FA) 0.6, apparent diffusion coefficient (ADC) 0.1, Axial diffusivity (AD) 0.2, radial diffusivity (RD) 0.06, and volume 7 mL

The means of MRI outcomes were as follows: CST FA 0.62 (± 0.02), ADC 0.08 (± 0.01), AD was 0.14 (± 0.01), RD was 0.05 (± 0.00) and volume was 7.76 mL (± 1.59) (example in Fig. 2).

TMS recordings were measured from the right FDI in 14 patients and from the left in 11 patients. The TMS measures were as follows: rMT 63.84 ± 14.37%, MEP latency 28.47 ± 5.30 ms, SICImean 28.62 ± 51.98%, SICImax 48.64 ± 40.36%, ICFmean 116.20 ± 148.90%, and ICFmax 174.60 ± 217.80%.

### Correlation analysis between TMS, MRI, clinical and demographic data

ICF_mean_ and ICF_max_ were significantly inversely correlated with RD (ρ =-0.56; *p* = 0.004 and ρ=-0.59; *p* = 0.002, respectively) and with ADC (ρ=-0.44; *p* = 0.030 and ρ=-0.45; *p* = 0.020, respectively). No significant correlations were observed between TMS measures and cortical lesions.

### Correlation analysis between TMS/MRI measurements and clinical data

ICF_mean_ was significantly and inversely correlated with disease duration (ρ=-0.46; *p* = 0.020). Also, MEP latencies were significantly directly correlated with EDSS scores (ρ = 0.55; *p* = 0.004). No other significant correlations were found between TMS/MRI data and sociodemographic/clinical data.

## Discussion

This work assessed the relationship between cortical excitability measures and neuroimaging measures of cortical lesions and corticospinal tract integrity, as well as clinical and demographic data in PwMS. We found that ICF measured by TMS was inversely correlated with RD and ADC of CST measured by MRI DTI. ICF was also inversely correlated with disease duration. Moreover, MEP latencies were directly correlated with EDSS scores. No significant correlations were found between cortical lesions and TMS measures.

To start, DTI is a technique that quantifies brain microstructure changes including inflammatory demyelination in MS. Different orientations of diffusion are acquired to measure water anisotropy within GM and WM with three eigenvectors (Basser et al., 1997). In white matter, the principal eigenvector (λ1) of the tensor aligns with the direction of the myelinated axon (Alexander et al. 2000). In mouse experiments, the AD defined by the principal eigenvector λ1 (i.e. the largest eigenvalue) was correlated with axonal loss, while the RD defined by the two other eigenvectors (λ2, λ3) was correlated with myelin content (Song et al. 2002, 2003). High ADC would reflect both demyelination and axonal pathologies. Besides DTI, the adopted TMS outcomes employed in our work (namely ICF and SICI) would reflect intracortical synaptic processes of facilitation and inhibition, respectively. ICF might increase in the early stages of the disease in an attempt to compensate for the ongoing pathological nervous system changes and prevent/minimize symptoms expression (Ayache and Chalah 2017). However, ICF could decrease with time which might reflect the depletion of the compensatory mechanisms, a finding that would be paralleled by disability progression (Ayache and Chalah 2017; Stampanoni Bassi et al. 2020).

In our study, ICF was inversely correlated with RD of CST. This correlation could be interpreted as follows: demyelinated axons within the CST would have an increased RD since the destruction of myelin facilitates the diffusion of water, perpendicular to the axon, through the absence of this structure. This phenomenon could be paralleled by a decreased ICF, reflecting worse synaptic plasticity. Conversely, preserved myelinated axons of CST would have a relatively low RD and a high ICF, compared to demyelinated ones which would be expected to exhibit the opposite. A previous study hints toward an increased RD in the CST of PwMS compared to healthy controls (Kern et al. 2011). In the same work, RD was the strongest DTI metric to reflect early demyelination and was found to correlate with clinical motor function measure (nine-hole peg test) (Kern et al. 2011).

ICF was also inversely correlated with ADC in our present work. Previous studies showed ADC was higher in PwMS compared to healthy controls (Tench et al. 2002; Lin et al. 2005; Cercignani & Gandini Wheeler-Kingshott, 2019). ADC would quantify the water diffusion along both axon and myelin (i.e., AD and RD). Consistently, in a previous work involving PwMS, ADC was correlated with pyramidal function system score (Lin et al. 2005).

As for AD, previous studies showed its impact on CST in PwMS since it was correlated with clinical measures of step length during walking performance (Hubbard et al. 2016) and with EDSS pyramidal score (Louapre et al. 2016). However, In our study, we did not find any significant correlation between AD and TMS measures. This absence of correlation might be linked to more specific myelin conduction evaluation by TMS rather than the axonal evaluation of CST. Another explanation would be a predominant myelin impairment compared to the relative preservation of axons as previously suggested (Roychowdhury et al. 2000), but this hypothesis could not be formally tested in the absence of histopathological correlation with MRI data.

Although no previous studies have evaluated the specific correlations between ICF/SICI with DTI/PSIR/DIR measures in PwMS, very few works have assessed the relationship between some neurophysiological and neuroimaging measures. For example, MS brain lesions were associated with high central motor conduction time (CMCT) (Cruz-Martínez et al. 2000; Kalkers et al. 2007; Madsen et al. 2022), reduced MEP amplitude (Madsen et al. 2022), and long cortical silent period (CSP) (Nantes et al. 2016b). Also, in the MS corpus callosum, DTI measures were correlated with transcallosal inhibition measured with MS (Lenzi et al. 2007; Wahl et al. 2011; Llufriu et al. 2012). Additionally, low neurite density was correlated with high motor cortex excitability (Radetz et al. 2021). Moreover, in one study using conventional MRI, prolonged CMCT by TMS was associated with spinal cord lesions (Kidd et al. 1998; Kalkers et al. 2007). Furthermore, the CSP was correlated with the volume of WM lesions adjacent to the primary motor cortex (Nantes et al. 2016b). Interestingly, they raised the question if this relationship was due to the WM lesion pathway leading to the cortex or cortical lesion since the applied method could not differentiate. In another work by the same authors, the correlation between cortical excitability measures and cortical magnetic transfer ratio varied according to the disease type (Nantes et al. 2016a).

In addition to the observed correlations concerning ICF, MEP latencies were also directly correlated with EDSS scores in this work in line with previous work involving PwMS (Mori et al. 2013). While ICF would reflect intracortical synaptic dysfunction, MEP latencies would reflect WM injury, and both were suggested as surrogate markers of disability in MS (Mori et al. 2013). Besides MS, cortical excitability measures are also gaining interesting in the field of other neuropsychiatric conditions such as stroke (Demirtas-Tatlidede et al. 2015; Julkunen et al. 2016) and schizophrenia (Mikell et al. 2016; Du et al. 2017).

It is important to state that motor cortex lesions did not correlate with TMS measures in the present work. This is probably due to the uncommon cortical lesion of this region, and the low number of patients with such lesions in the present work. Therefore, this could have led to insufficient statistical power. Further studies in MS would benefit from focusing on patients with documented motor cortical lesions in order to study the correlation between TMS measures and these cortical lesions.

It is noteworthy that this study had several limitations. First, our MS cohort was relatively small, and the results should be interpreted with caution. In fact, the small number of participants precludes any meaningful further comparisons, such as between the treated vs. untreated patient groups, or between the disease phenotypes (primary vs. secondary progressive MS). The current findings merits be reproduced in larger population. Second, DTI CST measures did not include cervical spinal cord part that would contribute to impaired TMS measures that might weaken the correlation between RD/ ADC with ICF. Third, only patients with progressive forms of MS were included since cortical lesions are more frequent in this form than relapsing-remitting form. Finally, admitting the cross-sectional and observation design of this work, future longitudinal works would help assess the evolution of the considered correlation.

## Conclusion

TMS measures of ICF was inversely correlated with RD/ADC of CST measured by DTI. Demyelination and aberrant synaptic plasticity might be reflected by concomitant structural and functional abnormalities as observed here. These outcomes might constitute reliable markers of disability and they merit further exploration.

## Data Availability

Data will be made available by the corresponding author upon reasonable requests.
